# Knowledge Gaps in Taxonomy, Ecology, Population Distribution Drivers and Genetic Diversity of African Sandalwood (*Osyris lanceolata* Hochst. & Steud.): A Scoping Review for Conservation

**DOI:** 10.3390/plants10091780

**Published:** 2021-08-26

**Authors:** Ben Belden Mugula, Samuel Kuria Kiboi, James Ireri Kanya, Anthony Egeru, Paul Okullo, Manuel Curto, Harald Meimberg

**Affiliations:** 1 School of Biological Sciences, College of Biological and Physical Sciences, University of Nairobi, Nairobi P.O. Box 30197-00100, Kenya; samuel.kiboi@uonbi.ac.ke (S.K.K.); jiykaya@uonbi.ac.ke (J.I.K.); 2 Department of Life and Physical Sciences, School of Natural Sciences, Bugema University, Kampala P.O. Box 6529, Uganda; 3 College of Environmental and Agricultural Sciences, Makerere University, Kampala P.O. Box 7062, Uganda; a.egeru@ruforum.org; 4 National Agricultural Research Organization (NARO), Entebbe P.O. Box 295, Uganda; paul.okullo@gmail.com; 5 Department of Integrative Biology and Biodiversity Research, Institute of Integrative Nature Conservation Research, University of Natural Resources and Life Sciences, Gregor Mendel-Straße 33, A-1180 Vienna, Austria; manuel.curto@boku.ac.at (M.C.); meimberg@boku.ac.at (H.M.); 6 MARE-Marine and Environmental Sciences Centre, University of Lisbon, Campo Grande, 1749-016 Lisbon, Portugal

**Keywords:** hemiparasites, molecular ecology, population genetics, conservation strategies

## Abstract

The increasing demand for ornamental, cosmetic and pharmaceutical products is driving exploitation of plant species globally. Sub-Saharan Africa harbours unique and valuable plant resources and is now a target of plant resource depletion. African Sandalwood (*Osyris lanceolata),* a multi-purpose and drought-tolerant species, has seen increased exploitation for the last thirty years and is now declared endangered. Initiatives to conserve *O. lanceolata* are not yet successful in Africa due to poor understanding of the species. This review surveys relevant research on the ecology, taxonomy, population dynamics, genetic diversity and ethnobotany of *O. lanceolata*, and highlights gaps in the literature for further research. A scoping review of grey literature, scholarly papers and reports was applied with pre-determined criteria to screen relevant information. Review findings indicate *O. lanceolata* is a globally distributed species with no identified center of origin. In Africa, it ranges from Algeria to Ethiopia and south to South Africa; in Europe it occurs in the Iberian Peninsula and Balearic Islands; in Asia from India to China, and also on Socotra. The species has a confusing taxonomy, with unresolved issues in nomenclature, country range distribution, extensive synonymisation and variation in growth form (shrub or tree). The species population is reported to be declining in Africa, but information on population dynamics across its entire range of distribution is anecdotal. Additionally, ecological factors influencing spatial distribution and survival of the species remain unknown. A variety of uses are reported for *O. lanceolata* globally, including: cultural; medicinal and food; dye; perfumery; timber; ethnoveterinary and phytoremediation. Key research areas and implications for conservation of *O. lanceolata* in Sub-Saharan Africa are proposed.

## 1. Introduction

The high global demand for ornamental, cosmetic and pharmaceutical products is driving exploitation of plant species all over the world [1]. Sub-Saharan Africa harbours an important stock of unique and valuable plant resources, and therefore is a target of expanding plant resource exploitation [2]. African sandalwood (*Osyris lanceolata Hochst. & Steud.*) is a multipurpose, drought-tolerant and hemiparasitic tree, well known for its essential oils used in perfumery industries [1]. It emerged as a potential commercial species in Africa due to significant decline in original sources of sandalwood oil, e.g., *Santalum album* L. (Indian subcontinent) and *Santalum spicatum* (R.Br.) A. DC. (Australia) in the 1990s, and the increasing demand for sandalwood oil over the years [3,4,5]. Dwindling of the species populations in Africa is attributed to overexploitation and lack of robust management strategies [5,6,7,8]. Some populations in Uganda, Kenya, Tanzania and South Sudan have completely disappeared due to illegal harvesting and smuggling of tree logs despite the species being protected under Appendix II of the Convention on International Trade in Endangered Species (CITES) [8,9,10,11]. *O. lanceolata* is assigned an automated status of least concern (LC) [12] with an unknown population trend but acknowledging decline in east Africa due to over exploitation [8,12].

Apparently, the lack of adequate information to reliably manage a sound resource base for *O. lanceolata* makes it very difficult to implement informed strategies for in situ and ex situ conservation in Africa [5]. Previous emphasis on plantations (ex situ strategy) and in situ measures for conservation have not succeeded due to information gaps on the species ecology, population dynamics and genetics [13,14,15]. Additionally, identification of suitable sources for genetic resource improvement is difficult without adequate scientific information on the species [15,16]. Knowledge of non-random distribution of genes from these studies may be even more important for conservation of the species [17,18]. Information on species population structure and demographic data help to predict the future stability of a species population amidst environmental and anthropogenic disturbances [19,20].

Whereas the ecology, population genetics and phylo-geography of other economically important species like *Prunus africana.* (Hook.f.) Kalkman. are documented in Africa [21,22], similar information is lacking for *Osyris lanceolata* [23]. There are peculiar ecological and genetic aspects of *O. lanceolata* which need to be understood and aligned with strategies for responsible management, in particular hemiparasitism, complex distribution patterns and low survival rate [24,25]. These broad attributes raise the following questions which require critical analysis: (i) What is the distribution, taxonomy and ethnobotany of *O. lanceolata*? (ii) Which environmental factors influence the species distribution and hemiparasitic relationships across habitats? and (iii) How do such factors impact on characteristics of the species population structure, genetic diversity and conservation status in Africa? Understanding these questions contributes to informed conservation strategies. 

This review analyses the missing links in population dynamics, ecology, taxonomy and genetic diversity of *Osyris lanceolata* using the available literature with a special interest in populations in Sub-Saharan Africa. We present the species taxonomy and ethnobotanical uses and discuss the role of hemiparasitism, while identifying emerging questions for further research. A global scope of the species distribution is provided and factors influencing its spatial distribution are explored. Further, we discuss the role of population structure assessment and general trends in the species population in Africa. Finally, the relevance of genetic diversity assessment, the extent of genetic studies on the species and implications for further research and conservation of *O. lanceolata* in Africa are proposed.

## 2. Methodology

### 2.1. Definitions

The key terms were defined to provide a scope of their meaning in this study [26,27] as follows: Population dynamics—changes in population structure (size class distribution), density, spatial distribution and abundance. Ethnobotany—different uses of *Osyris lanceolata*. Population distribution drivers—specific factors or conditions that favor distribution and survival of *O. lanceolata* in natural habitats. African sandalwood-*O. lanceolata* and its scientific synonyms.

### 2.2. Study Review Design

Research on African sandalwood is not yet extensive and thus a scoping approach was adopted to map the available literature [26,27,28]. The review process began with formulating a general research question as follows: “What is known in the literature about the ecology, taxonomy, population dynamics, hemiparasitism, ethnobotany and genetic diversity of *Osyris lanceolata*?” and which gaps exist in literature on the same topics? The following guiding questions were developed to explore the general question. (1) What is the distribution of *O. lanceolata* and its synonyms? (2) Which species are accepted in the genus *Osyris* and what are the synonyms for *O. lanceolata*? (3) Which ethnobotanical uses are reported for *Osyris lanceolata*? (4) What is the role of hemiparasitism in *Osyris* sp? (5) What are the suitable habitats, population trends and patterns of *O. lanceolata* across the range of distribution? (6) Which factors influence the species distributions? (7) Are there theories to explain distribution drivers for *Osyris* sp.? (8) What is the role of genetic diversity and to what extent has it been studied for *O. lanceolata* in Africa? (9) Which conservation implications could enhance informed strategies for responsible management of *O. lanceolata* in Sub-Saharan Africa?

#### Search Process

Relevant studies were identified through searching for evidence in electronic databases like SCOPUS, Web of Science and Google Scholar, websites, use of reference lists, manual searching of key journals, species taxonomy databases, USAID and CITES reports using the search terms: African sandalwood, *Osyris lanceolata*, Osyris species, plant species distribution drivers, population structure, population trends, Osyris taxonomy, hemiparasitism, reproductive biology and genetic diversity. Further information on genetic diversity was obtained through specific searches in molecular science journals. Specific papers were then sorted from different sources according to search topics and summarised into tables, figures and short paragraphs [26,27,28]. While analysing information, the main focus was on study aims, methods, findings, controversies, recommendations, conservation inferences and knowledge gaps for further studies.

## 3. Result of Search

We retrieved fewer studies on *Osyris* sp. compared to other sandalwood species such as *Santalum* sp. after conducting extensive searches on different study fields (see Table 1).

### 3.1. Taxonomy of Osyris sp.

Parasitic plants have been the most difficult plant groups to classify due to their specific adaptations in biology and morphology [46]. Most members of the genus *Osyris* are hemiparasitic plants, with complex physiognomy, physiology and morphology [24]. The genus belongs to the angiosperm order Santalales, family Santalaceae. Family Santalaceae has over forty genera and 400 species distributed in the tropics and temperate ecosystems [43]. The three African genera in family Santalaceae include *Thesium, Osyridicarpus* and *Osyris* [8]. Genus *Thesium* is the largest with over 200 species native to Africa and regions with a Mediterranean climate [8].

According to the webpage of the International Plant Names Index (www.ipni.org (accessed on 25 June 2021)) five species of the genus *Osyris* are accepted: *O. alba* L. with Mediterranean distribution in south Europe and north Africa, *O. daruma* Parsa with a range in southern Iran and *O. compressa* (P. J. Bergius) A. DC. and *O. speciosa* (A.W. Hill) J.C. Manning & Goldblatt, both with a main distribution in the Cap provinces. All remaining described species are not accepted to date and treated as one taxon, *Osyris lanceolata* Hochst. & Steud. (see Appendix A), resulting in a very large and inhomogeneous range for the species, with areas in south and east Africa, in parts of southern Europe and Asia. In the Catalogue of life (COL, www.catalogueoflife.org (accessed on 25 June 2021) the taxonomy that forms the base for the Global Biodiversity Information Facility (GBIF), and World Flora Online (WFO), which is a global online compendium of the world’s plant species, *O. lanceolata* is subdivided in *O. quadripartita* Salzm. ex Decne with the European and south and east African populations and *O. wightiana* Wall. ex Wight with the Asian populations (Appendix A). In this study, we use the name “*Osyris lanceolata*” in accordance with previous treatments of African sandalwood [44].

**Figure 1:**
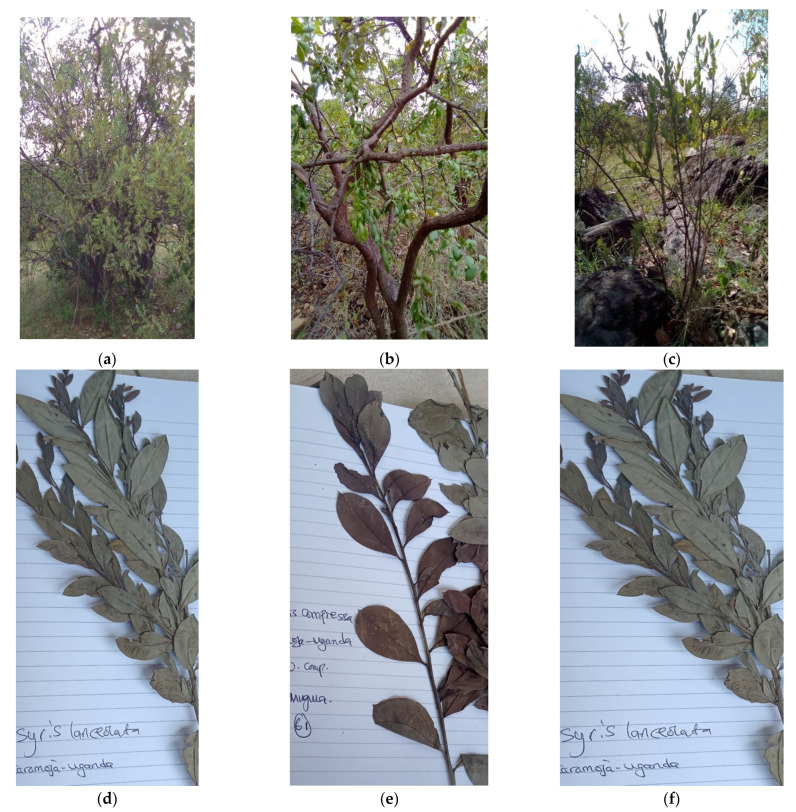
(**a**) Adult tree of *O.* *lanceolata* in Uganda; (**b**) “*O. compressa*” (adult tree); (**c**) *O. lanceolata* (sapling) [47]; (**d**) specimen of *O. lanceolata* from Karamoja; (**e**) “*O. compressa*” (Karamoja); (**f**) *O. lanceolata* (Karamoja) [47].

In consequence, the species *Osyris lanceolata* Hochst. & Steud. (1832) is represented by various synonyms [44,46] and multiple independent classifications [43]. The species (Figure 1) is commonly known as African sandalwood, east African sandalwood, Nepalese sandalwood, or false sandalwood. *O. lanceolata* is highly variable in morphology, especially leaf size [46] and shape depending on climate, altitude, edaphic variables and sex type [46], which may account for the occurrence of various synonyms. For instance, in some field work activities in Uganda, specimens of *Osyris lanceolata* were identified as *Osyris compressa* (Berg.) A. DC due to variations in leaf size and thickness leaves [47] as shown in Figure 1b,e. 

#### Global Distribution of *Osyris lanceolata*

**Figure 2:**
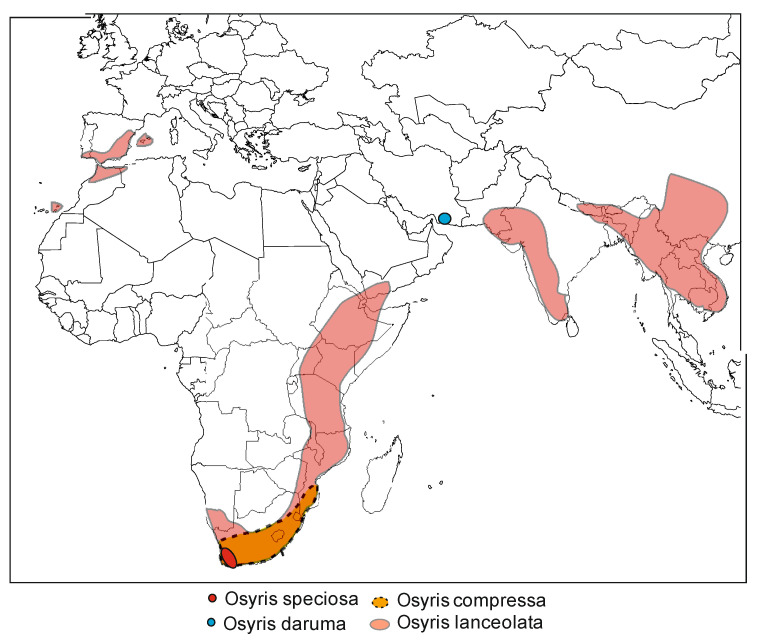
Global distribution of African–Asian Osyris species—*Osyris alba* with cross-Mediterranean distribution is not considered. Additional reports per country are given in the text.

We found evidence indicating a wide geographical distribution of *Osyris lanceolata* in Africa, Asia and parts of Europe [43,44,48], as indicated in Figure 2. In Africa, it ranges from Algeria to Ethiopia and south to South Africa; in Europe it occurs in the Iberian Peninsula and Balearic Islands; in Asia from India to China, and also on Socotra [44], but its center of origin is not currently known [8,49]. The native range of the species includes: Canary Islands in Spain, southern Iberian Peninsula, Baleares, Sahara to South Africa, Socotra, Indian subcontinent to south China and Indo-China [44,50]. Nevertheless, information from public databases like GBIF is prone to curation errors and, most importantly, it can be incomplete. For example, although not present in the GBIF database, there are reports indicating the occurrence of *O. lanceolata* in Tunisia, Libya and Egypt. Hence, there is a need for further exploration of the global distribution of this species.

As *Osyris* sp. occurs in diverse habitats, specific environmental variables influencing its distribution and population structure across isolated habitats are not known [25,51]. For instance, the Socotra Islands have been isolated from large landmasses for a long time which may have caused *Osyris* sp. to evolve multiple genetic lineages different from *Osyris* species in Africa. Similarly, *O. lanceolata* includes an island population on the Canary Islands. The distribution indicates the possibility of local adaptation of single populations. In addition, multiple lineages or even additional subspecies of sandalwood with distinct morphological and genetic characteristics could exist. Currently, available taxonomic solutions in the reviewed literature on the species do not reflect this complexity. Thus, characterising the genetic diversity and structure of populations from different regions to understand the origins of lineages within this widespread species is necessary to avoid translocations and artificial admixture and hybridisation effects due to commercial exploitation that may compromise conservation efforts, hence enhancing possible extinction of subpopulations with unique characteristics.

The country range distribution of *O. lanceolata* populations in Sub-Saharan Africa is shown in Table 2. Local distribution of populations for *O. lanceolata* synonyms is also reported as follows. *O. compressa* occurs in Uganda, *Osyris quadripartita* Decne. in Algeria, *Osyris abyssinica* A. Rich and *Osyris parvifolia* Baker. occur in Ethiopia, *Osyris pendula* Balf.f. is distributed on the Socotra Islands, *Osyris rigidissima* Engl. occurs in northern Somalia, while *Osyris tenuifolia* Engl., *Osyris oblanceolate* Peter., *Osyris laeta* Peter. and *Osyris densifolia* Peter. occur in Tanzania, and *Osyris urundiensis* De Wild occurs in Burundi. *Osyris quadrifida* Kirk. and *Osyris quadrifida* Salzm. ex DC. occur in Morocco. *Osyris quadrifida* Salzm and *Osyris quadripartita* Salzm. ex Decne. occur in Algeria [44].

We could not find a revision of the genus *Osyris* which described morphological variation and outlined potential subdivision into intraspecific taxa of the widely distributed species *O. lanceolata*. Synonymisation of described species had rather been carried out in regional revisions where the status of the material might not have been questioned or investigated in a wider context of the whole range of the species. This results in a situation that one species exists in very different areas, from the Mediterranean to the tropics, at high and low altitudes and in very different precipitation regimes. Even though it is possible that a species can be widely distributed by effective dispersal mechanisms, it is likely that individuals in the different hemispheres have been isolated from each other for long time and faced different selection pressures during long time spans. Thus, even if morphologically no species subdivision of *O. lanceolata* is suggested and no supporting characters can be found, very diverse adaptation patterns could exist in the species. For instance, analysis of genetic structure among members of the Ancistrocladaceae revealed multiple species in different ranges but having almost similar morphological variations that correspond to local adaptation [56,57]. This possibility is underlined not only by the large amount of different ecological conditions the species can be found in, but also life history traits like dioecy that can counteract colonisation, even though zoochory could provide a vector to connect distant populations.

The patchiness of the distribution in Kenya and Uganda indicates possible genetic differentiation of populations and a requirement of management of the species at a local scale and difficulties to restore locally extinct populations. Morphological variation and the diverse ecological conditions the species can occupy indicate that the current species circumscription underestimates species numbers. A close evaluation of morphology, ideally combined with molecular data, could suggest the recognition of additional taxa. The species that are poorly represented as herbarium specimens might be prone to underestimation of the real species diversity. One example is the Ancistrocladaceae where investigations on genetic structure showed the existence of multiple species with overlapping ranges, even though morphological variation did not allow a clear subdivision [56,57].

The possibility that multiple species could be recognised in *O. lanceolata* is underlined by recognition of different morphological forms. For instance, there is a general tendency to identify some east African sandalwood specimen as *Osyris compressa* (Figure 1b), when they show leaf characteristics which differ from the typical *O. lanceolata* habitus. However, it is unlikely that these specimens belong to *O. compressa* because they differ from this species in other characters and a critical examination of morphological characteristics is lacking. This highlights that morphological forms can be recognised and are used in the field but are not reflected by current taxonomic treatment. This calls for a revision and harmonisation of *Osyris* to resolve such anomalies in taxonomy, and avoid treatment of different species of *Osyris* as one species in experimental studies [33].

Molecular investigations can help to resolve confusion in the taxonomy of species with overlapping ranges, especially where morphological variations cannot provide an accurate basis for identification [56,57,58]. For instance, molecular and biochemical studies have proved to be of immense help to distinguish sandalwood oils obtained from different species (*Santalum* sp. vs. *Osyris lanceolata*/*Osyris weightiana*) [33]. Another example is when molecular data were used to differentiate the genus *Colpoon* from *Osyris* in South Africa [45]. Using morphological and phylogenetic data, it was established that the genus *Colpoon* is distinct from *Osyris*, and hence the two taxa are not congeneric, as was considered before. Resolving taxonomic uncertainty would help to conserve species diversity because exploitation can lead to overuse of specific populations and unnoticed extinction of parts of the diversity, hence decreasing the overall availability of the resource.

### 3.2. Biology of African Sandalwood: Hemiparasitism and the Missing Links

Plant parasitism is suggested to have evolved in arid environments where water and nutrients are scarce [59,60,61,62,63,64,65,66], to help parasitic plants access carbohydrates, water and mineral nutrients through hosts [60,61,62]. These hemiparasites make their own chlorophyll, but also need hosts to obtain water and nutrients to boost their survival. Without hosts, the hemiparasitic growth rate declines rapidly, especially in later stages due to poor acquisition of nutrients such as Ca^2+^, K^+^, P and Mg^2+^ [62,63,64,65,66]. For instance, seed germination of *Osyris lanceolata* does not require any host influence in the early stages [61,66] but further development of seedlings requires hosts [67]. A detailed analysis of the role of hosts on life stages of hemiparasites and mechanisms for interaction between hemiparasites and their hosts is already documented [67,68,69,70,71,72,73]. 

Some hemiparasites co-exist with a wide range of host species (over 440) [72] while others are extremely host specific [61,63,73]. Host specificity is not static but dynamic depending on levels of plant diversity within an ecosystem [60]. Thus, hemiparasites that are generalists across the entire range can be specific to particularly abundant hosts at a local scale [63,73]. For instance, a study in Tanzania observed common hosts for *Osyris lanceolata* in controlled environments to include: *Rhus natalensis*, *Dodonaea viscosa* Jacq., *Tecomaria capensis* (Thunb.), *Catha edulis* (Vahl) Forssk. Ex Endl., *Apodytes dimidiata* E.Mey. ex Arn., *Brachystegia spiciformis* Benth., *Maytenus acuminata* var. acuminata and *Aphloia theiformis* (Vahl) Benn. [37]. As hosts of hemiparasites differ geographically [23,63], it is also necessary to explore if this is due to their adaptation to local flora or because they are generalist plants. If they adapt to local flora, are there similar attributes in the biology of host plants in different regions of distribution? What role do such attributes play in the survival of hosts and hemiparasites? 

As hemiparasites rely on hosts to acquire nutrients, their survival is also tied to the degree of conservation of host plants [64,74]. However, hosts have other immediate socio-economic values, depending on the needs of local communities, hence the survival of hemiparasites becomes more fragile due to competing threats in the form of ethnobotanical or industrial plant uses. Secondly, the presence and absence of certain hosts will affect genetic diversity, and thus the evolutionary potential of a hemiparasite. If hemiparasites are generalists, then species composition may not have comparable influence on genetic diversity, but rather the main factors shaping their genetic diversity should depend upon the presence or absence of any hosts. Equally, if it is a specialist, its fitness will be dependent on the presence of certain key species and thus its diversity is also affected by their presence or absence. 

What is lacking in the biology of *Osyris lanceolata*, in particular, is an understanding of the factors influencing the use of available potential hosts at a local scale (host specificity), and the hierarchical ranking of host use (host preference). The identity of suitable hosts and their ecological roles in the survival of *O. lanceolata* at different stages of growth and development require further scrutiny. It is important to understand the trade-offs and variations in proximity levels between *O. lanceolata* and different hosts in natural habitats. This information is key in developing strategies for in situ and ex situ conservation. Further, we know much less about the factors influencing the distribution of hemiparasites and their hosts, and how host composition influences genetic diversity of hemiparasites [62,68]. Host abundance alone has been disputed to influence the distribution of hemiparasites, but rather there may be a combination of environmental factors interacting to determine definite distribution [60]. In addition, to narrow this gap in the ecological science of hemiparasites and enhance species conservation, a thorough investigation of the relationship between edaphic, biotic and other environmental factors in spatial distribution of *O. lanceolata* in natural populations is necessary. These analyses could improve understanding of suitable conditions for species survival and adaptation to enhance conservation strategies.

#### Genetic Studies on African Sandalwood

We found a handful of genetic studies on *Osyris lanceolata* unlike other species such as *Santalum* sp. For instance, we found three studies focused on the genetics of *O. lanceolata* [33,34,35]. Two studies [34,35] were focused on developing microsatellite markers to assess genetic diversity of *O. lanceolata* in the Kenyan populations. The study [35] identified and developed 12 polymorphic and five monomorphic markers for population genetic studies including assessment of gene flow levels in different populations. The second study by Andiego [34] assessed genetic diversity and population genetic differentiation among seven populations in Kenya. As a result, the most genetically distinct populations were identified for conservation strategies. In this case, assessment of genetic diversity is crucial in identification of populations for conservation priority and creating baseline data for informed conservation strategies at the local scale. Such decisions cannot be made without genetic data on species populations. However, these studies have not been carried out on other populations in east Africa which creates a huge gap in the knowledge on the species. Another study focused on identification of *Santalum album* and *O. lanceolata* using biochemical characteristics and molecular markers to check adulteration in Asia [33]. This study highlighted the importance of using biochemical characteristics and nucleotide sequence dissimilarities in the rRNA genes to distinguish between *Santalum alba* and *O. lanceolata*, and also provided a molecular framework and methodology for checking adulterations in sandalwood oils.

The limited number of studies on genetics of *Osyris lanceolata* creates a huge gap in the understanding of the genetic adaptive potential of the species in Africa, given the changing environmental conditions affecting the survival of the species. Analysis of genetic diversity involves assessment of genetic variation in time and space, to understand the species dispersal, mating behavior, delimitations and population boundaries [75]. This helps to obtain information about the species population structure and degree of connectivity and identify barriers to gene flow within and among populations [76] so as to design informed strategies for conservation. High genetic diversity provides more alleles to increase genetic adaptive potential and fitness of populations in response to environmental changes [77].

Unlike habitat loss and fragmentation which can have an extreme impact on genetic diversity of plant species [77,78,79], domestication was established to have minimal impacts on plant genetic diversity in the short term [76,77,78,79,80]. Despite this assertion, we did not find studies to show how asexual reproduction (through ) and propagation influences the genetic diversity of *O. lanceolata*. On the contrary, human activities were reported not to impact genetic diversity of species such as *Scaphium macropodum* (Miq.) Beumee ex. K. Heyne., *Dryobalanops aromatica* C.F.Gaertn. and *Shorea curtisii* Dyer ex. King. across generations [81]. Although regeneration of *O. lanceolata* is more successful through coppicing or root stocks, rather than seed germination [7], the mother source which contains higher genetic diversity in subsequent generations is not known. The factors accelerating seed germination failure in *O. lanceolata* need to be explored further to improve recruitment programs in natural populations. Thus, further studies to understand variations in genetic diversity across life stages, and between asexually produced individuals (root stocks/sucker/coppicing) and sexually propagated individuals of *O. lanceolata* are necessary to guide conservation actions such as restoration of overexploited habitats.

Genetic diversity assessment is also necessary to forecast changes in genetic structure and document loss of genetic diversity in populations of plant species [81,82,83]. Understanding fine-scale spatial genetic structure helps to describe the non-random distribution of genotypes in space within populations due to genetic drift, selection and gene flow [84,85]. It also helps to detect gene dispersal distance and the extent to which ecosystem disturbance can influence non-random distribution of genes in a population, leading to inbreeding and loss of genetic diversity [86]. Based on tremendous advancements in genetic technology [87,88,89,90,91,92,93,94,95,96,97,98,99], future studies on *O. lanceolata* should consider this focus, to evaluate populations and their suitability as provenances for in situ conservation, commercial propagation, restoration and further genetic improvement of the species.

### 3.3. Ecology: Habitats and Drivers for Distribution of African Sandalwood

*O. lanceolata* occurs in a diverse range of habitats including upland dry evergreen forests and mist forests characterised by bushland and grassland that usually extend downwards to rivers and slightly into deciduous woodlands at 900–2700 m above sea level [100]. Other suitable habitats for *O. lanceolata* include: dry savanna forests and woodlands, moist woodlands, thicket edges and dry submontane *Hyparrhenia* grasslands at an elevation range of 1000 m to 1730 m above sea level [101]. However, the species also occurs in rocky and non-rocky habitats [102,103] at even higher altitudes ranging from 900 m to 2250 m and with mean annual rainfall of 600 to 1600 mm with well-drained soils, but it cannot tolerate frost conditions [103].

Despite the reported habitats for the species, little is known about suitable survival conditions, and factors that would influence the species distribution in natural habitats. Understanding plant species distribution drivers helps to analyse the species survival conditions and strategies in habitats which is important in conservation planning. Although scientific evidence suggests *Osyris lanceolata* exhibits a clumped or patchy distribution [66], little is known about drivers of the species distribution. *O. lanceolata* is typically rare throughout its distribution range and also has a non-uniform pattern of distribution even in areas with abundant suitable hosts [66]. The highly patchy nature of distribution clearly suggests the influence of specific factors in determining the species distribution. A recent study by Fox [100] proposes the presence of hosts and habitat attributes as key determinants for the distribution of parasitic plants.

In other studies, the importance of habitat quality [101,102] is stressed, while seed dispersal capability could also influence species distribution [103,104]. Host quality includes water availability, edaphic variables and nutrients [66]. According to the host quality hypothesis (HQH) *O. lanceolata* can only establish and grow if they parasitise a host with sufficient quality such as one with low water stress [66]. In areas where water is limited, parasitic plants are likely to establish on hosts with greater access to water [66]. Proper illustration of this hypothesis requires a detailed field assessment of habitat quality for *O. lanceolata* populations and their hosts in natural populations. In addition, the abundant center hypothesis (ACH) suggests that a species will be more abundant where conditions for reproduction and population growth are most suitable [101]. A further implication of this hypothesis is that population density of a species declines towards areas with less suitable environments [101]. Therefore, if the spatial distribution of a species is correlated with corresponding environmental variables, an insight into drivers of species distribution and survival can be obtained as an indication of desirable survival conditions of a species in natural environments. Although we found studies suggesting specific germination requirements, seed vectors and site–microsite preference [66,101,104,105,106] as key drivers for species distribution, these conditions cannot account for the highly patchy spatial structure of *O. lanceolata* [66]. Thus, empirical data are required to understand key drivers of the distribution of *O. lanceolata* in natural habitats.

#### 3.3.1. Population Dynamics of *Osyris lanceolata* in Sub-Saharan Africa

We found overall support in the literature for a declining trend in populations of *Osyris lanceolata* which is believed to be endangered in Africa due to overexploitation [6,8,11,107], habitat loss [105,106] and accidental destruction of host plant species for fuel wood, timber, charcoal burning and building materials [47]. For instance, the species is smuggled by uprooting the whole plant in Uganda and Kenya, hence leading to loss of genetic variability and population decline [8]. Habitat loss is fueled by human activities such as deforestation, urbanisation, logging and mining, leading to land cover change, conversion and land use intensification with eventual loss of ecosystem services [105,106]. Other activities threatening *O. lanceolata* habitats include overgrazing and bush burning. The loss of natural habitats reduces local species abundance and diversity which leads to population decline and extinctions [105].

The lack of informed conservation strategies in Africa also exacerbates the decline in *Osyris lanceolata* populations [106]. With the rapid depletion of tropical forests, over 125,000 km^2^ per year [105], which form a significant portion of the habitats for *O. lanceolata*, and lack of informed conservation measures, urgent action is needed to save the species from total depletion. Some African governments have responded to address overexploitation by instituting restrictive policy actions such as presidential decrees prohibiting trade in the species products (Kenya, Uganda, Tanzania), sanctions on illegal traders and by-laws [8]. These are commendable practices, but further strategies are needed to promote sustainable harvesting, production and conservation in Africa.

To establish robust management strategies for *Osyris lanceolata*, studies to generate scientific data on the species population status and genetics are necessary. The lack of empirical data makes it difficult to design measures for effective species management, including monitoring population trends of *O. lanceolata* in Africa. Assessment of a species population structure helps to detect reasons for population decline, threats and human impact on species genetic diversity which is necessary to guide habitat management responses for declining species [108,109,110,111]. Secondly, information on population structure complements genetic studies towards understanding the interaction between evolutionary processes and environmental forces in shaping species adaptation in ecosystems [74]. Additionally, comparing demographic data with genetic diversity data helps to assess threats and identify genetically diverse populations of plant species [6]. 

#### 3.3.2. Ethnobotany of African Sandalwood

Documented uses of *Osyris lanceolata* can be divided into categories that include: cosmetics, emergency food, pharmaceutical industries, crafts, cultural/spiritual uses, local medicine, timber and ecological services (phytoremediation) [4,8,54,112,113,114,115,116,117,118], as summarised in Table 3.

We found extensive uses attached to *Osyris lanceolata* around the world, with essential oils being the most commercially valuable and tradeable resource [4,5]. The oils are naturally contained in the bark, lower stem and roots of sandalwood species, they contain α, β and epi-β-santalols as active ingredients [112] and are used in the production of perfumes, toiletries, mouth fresheners, incense, cosmetics, aromatherapy [112] and flavoring agents [4]. The oils are reported to have blending and antiseptic properties suitable for making fixatives in other fragrances [117]. The same oils have chemo-preventive properties used to manage eruptive and inflammatory skin diseases [118]. Other diseases such as dysuria, bronchitis and gonorrhoea can be treated with sandalwood oils [119]. *O. lanceolata* products are used to treat candidiasis [32], malaria [53], diarrhoea [54,120], chest pain and fever in Africa [112,113,114,115,116,117,118,119,120,121,122]. The oil and wood are burnt during spiritual and cultural ceremonies by Muslims, Hindus and Buddhists [4,122]. The bark and roots provide a red dye for skin tanning [78] while its shoots provide antipyretic agents for cattle in Africa [114]. The root system can be used to accumulate heavy metals and is hence useful in phytoremediation strategies [116]. Irrespective of sandalwood species, the major tradable products include oil, powder and wood logs, and these have significant markets in Germany, the United Kingdom, France, South Africa, the United States, India, the United Arab Emirates and China [4,5,8].

Although a large variety of uses for *Osyris* sp. are reported, the majority of studies cover medicinal or pharmaceutical and perfumery uses of the species. Only a handful of studies focus on other uses such as phytoremediation and ethnobotanical uses. Among medicinal uses, limited studies were focused on assessing the efficacy of concoctions from *Osyris* species in the treatment of human and veterinary diseases. There is need to document detailed ethnobotanical uses and indigenous knowledge associated with *Osyris* species so as to guarantee conservation of traditional knowledge on the species. Understanding the multiple alternative and local uses of a slow-growing species such as *Osyris lanceolata* helps to improve the attitudes of local communities towards conservation of that species [19]. These communities can derive socio-cultural and ecological benefits from the species in the short term, in addition to economic benefits which could be gained later if sustainable populations are conserved.

### 3.4. Implications for Conservation of Osyris lanceolata in Sub-Saharan Africa

This paper highlights four major issues with significant implications for conservation of *O. lanceolata* in Africa. First, the taxonomy of *O. lanceolata* is still complex (see Table A1) due to over synonymisation, country range distribution and ambiguity in species ranking. Second, the population dynamics of *O. lanceolata* across its range of distribution are anecdotal, though CITES reports indicate significant population declines, particularly in east Africa due to overexploitation. Third, the drivers of the spatial distribution of *O. lanceolata* in natural habitats are not understood. The species is highly patchy and exhibits an irregularly clustered pattern of spatial distribution which requires further analysis. Fourth, the species genetic diversity and ethnobotany are barely studied and hence not understood. These issues affect conservation of *O. lanceolata* as follows: the confusion in the taxonomy of *O. lanceolata* leads to continuous treatment of different species of *Osyris* as one taxon which may lead to loss of unnoticed populations with diverse morphological and genetic attributes. Secondly, continuous harvesting and utilisation of *O. lanceolata* with unknown population dynamics puts the species at a greater risk of depletion since the absence of population data complicates species monitoring and management. In addition, poor understanding of drivers of the distribution of *O. lanceolata* is a hindrance to conservation in Africa. Drivers of spatial distribution correlate strongly to suitable conditions for survival and fitness of a species in natural habitats and hence such information is necessary in planning for conservation approaches. Additionally, limited understanding of the genetic diversity of the species and structure hinders conservation efforts. For instance, suitable provenances cannot be identified easily to boost conservation programs. Equally, limited documentation of the ethnobotanical uses of the species also hinders conservation initiatives. Local communities may be reluctant to appreciate conservation of a species whose value and benefits are not understood.

The three approaches needed for continued survival of *O. lanceolata* populations include: conservation, restoration and sustainable commercial use. In particular, conservation of threatened habitats for the species population is necessary [123]. As different populations exhibit different population dynamics, conservation planning ought to be undertaken at the population level and reinforced by local investigations which are more informative than global studies [124]. Additionally, locally adapted monitoring protocols that consider different stakeholders at local and regional levels are key in tracking populations of threatened species [125]. However, these actions cannot be realised without adequate scientific information as a basis for informed policy actions. Finally, we emphasise that the risk of extinction of a species without adequate scientific data is high and impacts are extreme if resource extraction continues without planned strategies. Thus, our findings will stimulate constructive debate and more focused research towards responsible management of *Osyris lanceolata* in the long run, to avert the looming threat of extinction of the species in Sub-Saharan Africa.

## 4. Conclusions

The purpose of this study was to survey relevant research on the taxonomy, ecology, population dynamics, ethnobotany and genetic diversity of *Osyris lanceolata*, and highlight knowledge gaps for further research. We established that *O. lanceolata* is distributed in Africa, Asia, Europe and the Socotra Islands with no identified center of origin. The species has a relatively confusing taxonomy, with unresolved issues in nomenclature, country range distribution, oversynonymisation and uncertainty in biological form (shrub or tree), which calls for a deliberate global revision and harmonisation to resolve anomalies in taxonomy. Information on the species population dynamics across its entire range of distribution is anecdotal. Secondly, several use categories are reported for *O. lanceolata*. There are a handful of studies on the genetics and ecology of *O. lanceolata* in Africa. The available studies help little to understand the underlying factors for the species distribution and its survival in natural habitats. There are no scientific data to explain how the species genetic diversity varies across life stages and between modes of propagation (seed and asexual). Our review suggests that, currently, (i) species distribution drivers which are possible factors for survival of *O. lanceolata* in natural populations are invariably barely studied and (ii) despite the vital role of genetic diversity assessment in the conservation of plant genetic resources, and the availability of molecular techniques for its investigation, it is the least studied area for *O. lanceolata*, which partly underpins the slow progress in improvement in the species and its conservation in Africa. Therefore, a deliberate focus to understand detailed ethnobotanical uses and the ecological, population dynamics and genetic characteristics of *O. lanceolata* is urgently needed in present and future studies to enhance informed strategies for sustainable management of the species in Africa.

## Figures and Tables

**Table 1:** Summary of search results on sandalwood species.

Search Topic	Google Scholar		Web of Science		Scopus		Screened Papers			Author(s)
	*Santalum* sp.	*Osyris* sp.	*Santalum* sp.	*Osyris* sp.	*Santalum* sp.	*Osyris* sp.	General Papers	*Santalum* sp.	*Osyris* sp.	*Osyris* sp.
Population dynamics	4760	833	2250	11	347	50	17	07	06	[7,8,25,29,30,31]
Ethnobotanical uses	-		06	01	71	18	16	11	05	[3,29,30,31,32]
Genetic diversity	304.8	538	70	02	101	15	43	07	03	[33,34,35]
Hemiparasitism	-	-			01	00	22	04	03	[24,36,37]
Distribution drivers	-	-	00	01	-	-	09	-	03	[38,39,40]
Propagation methods	-	-			44	11	-	-	02	[41,42]
Reproductive biology	4030	01	01	393			-	-	01	[16]
Species taxonomy	-	-	12	01	125		32	-	05	[29,30,43,44,45]

**Table 2:** Country range distribution of Osyris lanceolata in Sub-Saharan Africa.

African Region	Country Ranges	Local Populations (Reported)
East Africa	Uganda, Kenya, Tanzania, Rwanda, Burundi, South Sudan	Uganda: Karamoja subregion, Mbale (Kaburorun), West Nile, Bukwo, Kween and Kapchorwa [8,44,47] Kenya: Turkana, Baringo, Bogoria, Narok, Amboseli, Pokot, Samburu, Laikipia, Kajiado, Kitui, Taita hills, Chyulu hills, Gwasi hills, Marsabit, Makueni, Kikuyu escarpment forest, Mbeere, Narok, Ol-donyo Sabul, Oloitokitok and Mt. Kulal [8,38,44,52,53,54,55] Tanzania: Ufipa, Mbulu district, Mbisi, Lake Manyara, Songea, Ihang’ana forest, Kilimanjaro region, Masai Boma, Oldoinyo Sambu [7,30,41,44,52] Rwanda: Akagera, Eastern Province [9,44] Burundi: Near Lake Shohoho and Rugweru region [44]
Southern Africa	Namibia, Zambia, Zimbabwe, Mozambique, South Africa, Malawi, Swaziland, Botswana	South Africa: Eastern Cape; Free state, Gauteng, Kwazulu-Natal [7,44,52]
Central Africa	Chad, Lesotho	Not reported in literature
North Africa	Algeria, Ethiopia, Somalia, Morocco, Tunisia, Gibraltar	Ethiopia: Shoa, Domak, Efat, Roth, Adua, Mt. Sholoda, Tigray [30] Somalia: Surud, Mt. Maydh, Mt. Hildebrandt [30] Algeria: Tangiers [8,44]
Cross-border Islands	Socotra	Socotra: Yemen, Haghier Hills [44]

**Table 3:** Ethnobotanical uses of *Osyris lanceolata*.

Use Category	Plant Resource	Description
Cultural/spiritual uses/perfumery	Wood and oil	Oils are used to make perfumes and cosmetics [112] while the wood is burnt during ceremonies in Asia [4]
Pharmaceutical/local medicine	Leaves, bark, root	Oils are used in pharmaceutical industries [112], local decoctions to treat malaria [53] and for sexually transmitted diseases (STDs) [32], chest pain, hepatitis B, fever, diarrhoea, chronic mucus infections, cough and asthma [113]
Tanning and dyeing	Roots	Roots contain a red dye used for tanning leather in Africa [114]
Food	Root, bark oil extract; fruits	Roots and bark provide flavored powder for tea and are used as tonics The root extract is used in preservation of milk in Africa, while the fruits are eaten as emergency foodstuffs by children and herdsmen in east Africa [113,114,115]
Ecological services	Root system	Root haustoria can accumulate heavy metals for phytoremediation [1,116]
Timber	Wood	Hardwood is used to make carvings and fencing for homesteads in Africa [5,8]
Ethnoveterinary uses	Leaves	The leaves are used as fodder, and also contain antipyretic agents for cattle in east Africa [117]

## Data Availability

All data generated or analyzed during this study are included in this article.

## Appendix A

**Table A1:** Summary of accepted species(genus Osyris) and synonyms in the treatments of IPNI, COL, WFO and Tropicos. COL does not accept O. lanceolata but divides it into O. quadripartita and O. wightiana.

Species	Synonym IPNI	Synonym COL	Synonym WFO	Synonym Tropicos
*Osyris* L.				
*Osyris alba* L.				
		*Osyris mediterranea* Bubani	*Osyris mediterranea* Bubani	*Osyris mediterranea* Bubani
*Osyris compressa* (P.J. Bergius) A. DC.				
*Osyris daruma* Parsa				
*Osyris speciosa* (A.W. Hill) J.C. Manning & Goldblatt				
*Osyris lanceolata* Steud. & Hochst. ex A.DC.				
	*Osyris quadripartita* Salzm. ex Decne.		*Osyris quadripartita* Salzm. ex Decne.	*Osyris quadripartita* Salzm. ex Decne.
		*Osyris abyssinica* Hochst. ex A. Rich.	*Osyris abyssinica* Hochst. ex A. Rich.	*Osyris abyssinica* Hochst. ex A. Rich.
		*Osyris densifolia* Peter, Fedde, Repert	*Osyris densifolia* Peter	*Osyris densifolia* Peter
		*Osyris laeta* Peter, Fedde, Repert.	*Osyris laeta* Peter	*Osyris laeta* Peter
		*Osyris oblanceolate* Peter	*Osyris oblanceolate* Peter	*Osyris oblanceolate* Peter
		*Osyris parvifolia* Baker	*Osyris parvifolia* Baker	*Osyris parvifolia* Baker
		*Osyris pendula* Balf.f	*Osyris pendula* Balf.f	
		*Osyris quadrifida*		
		*Osyris quadripartita* var.		*Osyris quadripartita* var. *puberula* Kumari
		*Osyris rigidissima* Engl.	*Osyris rigidissima* Engl.	*Osyris rigidissima* Engl.
		*Osyris tenuifolia* Engl.	*Osyris tenuifolia* Engl.	*Osyris tenuifolia* Engl.
		*Osyris urundiensis* De Wild	*Osyris urundiensis* De Wild.	*Osyris urundiensis* De Wild.
	*Osyris wightiana* Wall. ex Wight		*Osyris wightiana* Wall. ex Wight	*Osyris wightiana* Wall. ex Wight
		*Osyris divaricata* Pilg		
		*Osyris arborea* Wall.	Osyris arborea wall. ex A. DC.	Osyris arborea wall. ex A. DC.
		*Osyris nepalensis* Griff	*Osyris nepalensis* Griff	
		*Osyris wightiana* var. puberula (Hook.fil.)		
		*Osyris wightiana* Wall.	*Osyris wightiana* var. rotundifolia (P.C. Tam)	*Osyris wightiana* var. rotundifolia (P.C. Tam) P.C. Tam
			*Osyris arborea* var. rotundifolia P.C. Tam	*Osyris arborea* var. rotundifolia P.C. Tam
			*Osyris arborea* var, spitata Lecomte	*Osyris arborea* var, spitata Lecomte
			*Osyris abyssinica* var. speciosa A.W. Hill	*Osyris abyssinica* var. speciosa A.W. Hill
			*Osyris wightiana* var. spitata (Lecomte) P.C. Tam	*Osyris wightiana* var wightiana
				*Osyris spinescens* Mart. & Eichler
				*Osyris peltate* Roxb.

Source: [29,30,32,33,126,127]. IPN: International Plant Names Index; COL: Catalogue of Life; WFO: World Flora Online; Tropicos: Online botanical database.

## References

[1] da Silva J.A.T., Kher M.M., Soner D., Nataraj M., Dobránszki J., Millar M.A. (2018). Santalum molecular biology: Molecular markers for genetic diversity, phylogenetics and taxonomy, and genetic transformation. Agrofor. Syst..

[2] Ola O., Benjamin E. (2019). Preserving Biodiversity and Ecosystem Services in West African Forest, Watersheds, and Wetlands: A Review of Incentives. Forests.

[3] Mbuya L.P., Msanga H.P., Ruffo C.K., Birnie A., Tengnas B.O. (1994). Useful Trees and Shrubs for Tanzania.

[4] Page T., Hannington T., Bunt C., Potrawiak A., Berry A. (2012). Opportunities for the Smallholder Sandalwood Industry in Vanuatu.

[5] Thomson L.A.J. (2020). Looking ahead—Global sandalwood production and markets in 2040, and implications for Pacific Island producers. Aust. For..

[6] Rao M.N., Ganeshaiah K.N., Shaanker R.U. (2007). Assessing Threats and Mapping Sandal Resources to Identify Genetic ‘Hot-Spot’for In-Situ Conservation in Peninsular India. Conserv. Genet..

[7] Mwang’Ingo P.L., Teklehaimanot Z., Lulandala L.L., Mwihomeke S.T. (2005). Host plants of *Osyris lanceolata* (African Sandalwood) and their influence on its early growth performance in Tanzania. S. Afr. For. J..

[8] CITES (2013). Consideration of Proposals for Amendment of Appendices I and II.

[9] Muhoozi J. Encroachers Target Kabaruka in Akagera. The East African. http://www.theeastafrican.co.ke/rwanda/News/Encroachers-target-Kabaruka-in-Akagera/1433218-2093116-sy2phc/index.html.

[10] Tajuba P. How Oil Firm Is Burning Up Karamoja Valuable Trees. http://www.monitor.co.ug/artsculture/Reviews/oil-firm-burning-up-Karamoja-valuable-trees/691232-2996318-r5xq40/index.html.

[11] Bunei E.K. (2017). The Hunt for the Precious Wood: Illegal Trade of Sandalwood as an International Criminal Enterprise in Kenya. Soc. Bus. Rev..

[12] Wilson B. *Osyris lanceolata*. The IUCN Red List of Threatened Species 2018: e.T200642A2675362. https://www.iucnredlist.org/species/200642/2675362.

[13] USAID (2015). Uganda Environmental Threats and Opportunities Assessment (Etoa) Final Etoa.

[14] Uganda Investment Authority (UIA) (2016). Karamoja Investment Profile.

[15] Neuhauser C., Andow D.A., Heimpel G.E., May G., Shaw R.G., Wagenius S. (2003). Community Genetics: Expanding the Synthesis of Ecology and Genetics. Ecology.

[16] Mwang’ingo P.L., Teklehaimanot Z., Hall J.B., Zilihona J.E. (2007). Sex Distribution, Reproductive Biology and Regeneration in the Dioecious Species *Osyris lanceolata* (African Sandalwood) in Tanzania. Tanzan. J. For. Nat. Conserv..

[17] Kalisz S., Nason J.D., Hanzawa F.M., Tonsor S.J. (2001). Spatial Population Genetic Structure in Trillium Grandiflorum: The Roles of Dispersal, Mating, History, and Selection. Evolution.

[18] Curto M., Nogueira M., Beja P., Amorim F., Schümann M., Meimberg H. (2015). Influence of past agricultural fragmentation to the genetic structure of Juniperus oxycedrus in a Mediterranean landscape. Tree Genet. Genomes.

[19] Tabuti J.R.S., Mugula B.B. (2007). The ethnobotany and ecological status of *Albizia coriaria* Welw. Ex Oliv. In Budondo Sub-county, eastern Uganda. Afr. J. Ecol..

[20] Virillo C.B., Martins F.R., Tamashiro J.Y., Dos Santos F.A.M. (2011). Is size structure a good measure of future trends of plant populations? An empirical approach using five woody species from the Cerrado (Brazilian savanna). Acta Bot. Bras..

[21] Muchugi A., Lengkeek A.G., Kadu C.A.C., Muluvi G.M., Njagi E.N.M., Dawson I.K. (2006). Genetic variation in the threatened medicinal tree *Prunus africana* in Cameroon and Kenya: Implications for current management and evolutionary history. S. Afr. J. Bot..

[22] Kadu C.A.C., Parich A., Schueler S., Konrad H., Muluvi G.M., Eyog-Matig O., Muchugi A., Williams V., Ramamonjisoa L., Kapinga C. (2012). Bioactive constituents in *Prunus africana*: Geographical variation throughout Africa and associations with environmental and genetic parameters. Phytochemistry.

[23] Qasem J.R. (2006). Host range of the parasitic weed *Osyris alba* L. in Jordan. Weed Biol. Manag..

[24] Nurochman D., Matangaran J.R., Santosa G., Suharjito D., Sari R.K. (2018). Autecology and morphological properties of sandalwood (*Santalum album*) in Pidie District, Aceh, Indonesia. Biodivers. J. Biol. Divers..

[25] Gathara M., Makenzi P., Kimondo J., Muturi G. (2014). Prediction of *Osyris lanceolata* (Hochst. Steud.) Site Suitability Using Indicator Plant Species and Edaphic Factors in Humid Highland and Dry Lowland Forests in Kenya. J. Hortic. For..

[26] Munn Z., Peters M.D.J., Stern C., Tufanaru C., McArthur A., Aromataris E. (2018). Systematic review or scoping review? Guidance for authors when choosing between a systematic or scoping review approach. BMC Med. Res. Methodol..

[27] Arksey H., O’Malley L. (2005). Scoping studies: Towards a methodological framework. Int. J. Soc. Res. Methodol..

[28] Lauwers L., Bastiaens H., Remmen R., Keune H. (2020). Nature’s Contributions to Human Health: A Missing Link to Primary Health Care? A Scoping Review of International Overview Reports and Scientific Evidence. Front. Public Health.

[29] (2021). *Osyris lanceolata* Hochst. & Steud. In GBIF Secretariat.

[30] (2019). The International Plant Names Index Collaborators. International Plant Names Index.

[31] Mwang’ingo P.L., Teklehaimanot Z., Hall J.B., Lulandala L.L.L. (2003). African Sandalwood (*Osyris lanceolata*): Resource Assessment and Quality Variation among Populations in Tanzania: Research Note. S. Afr. For. J..

[32] Masevhe N.A., McGaw L.J., Eloff J.N. (2015). The traditional use of plants to manage candidiasis and related infections in Venda, South Africa. J. Ethnopharmacol..

[33] Bhat K.V., Balasundaran M., Balagopalan M. (2006). Identification of Santalum Album and Osyris Lanceolata through Morphological and Biochemical Characteristics and Molecular Markers to Check Adulteration.

[34] Andiego K.P., Dangasuk O.G., Odee D.W., Omondi F.S., Otieno D.F., Balozi B.K. (2019). Genetic diversity of endangered sandalwood (*Osyris lanceolata*) populations in Kenya using ISSR molecular markers. East Afr. Agric. For. J..

[35] Otieno J.O., Omondi S.F., Perry A., Odee D.W., Makatiani E.T., Kiplagat O., Cavers S. (2016). Development and characterization of microsatellite markers for *Osyris lanceolata* Hochst. & Steud., an endangered African sandalwood tree species. Trop. Plant Res..

[36] Newbold T., Hudson L.N., Hill S.L.L., Contu S., Lysenko I., Senior R., Borger L., Bennett D.J., Choimes A., Collen B. (2015). Global effects of land use on local terrestrial biodiversity. Nature.

[37] Mwang’Ingo P.L., Teklehaimanot Z., Maliondo S.M., Msanga H.P. (2004). Storage and pre-sowing treatment of recalcitrant seeds of Africa sandalwood (*Osyris lanceolata*). Seed Sci. Technol..

[38] Wambua J.K. (2010). The Distribution, Abundance and Ecological Impacts of Invasive Plant Species at Ol-Donyo Sabuk National Park, Kenya. Ph.D. Thesis.

[39] Erbo K., Tolera M., Awas T. (2020). Distribution, Association and Population Structure of Osyris Quadripartita (African Sandalwood) in a Dry Woodland Forest, Southern Ethiopia. Glob. J. Biol. Agric. Health Sci..

[40] Abascal F., Corvelo A., Cruz F., Villanueva-Cañas J.L., Vlasova A., Marcet-Houben M., Martínez-Cruz B., Cheng J., Prieto P., Quesada V. (2016). Extreme genomic erosion after recurrent demographic bottlenecks in the highly endangered Iberian lynx. Genome Biol..

[41] Teklehaimanot Z., Mwang’Ingo P.L., Mugasha A.G., Ruffo C.K. (2004). Influence of the origin of stem cutting, season of collection and auxin application on the vegetative propagation af African Sandalwood (*Osyris lanceolata*) in Tanzania. S. Afr. For. J..

[42] Kamondo B., Juma B., Mwangi L., Meroka D. (2007). Domestication of Osyris lanceolata in Kenya: Propagation, Conservation, Management and Commercialisation.

[43] Osyris lanceolata Hochst *Osyris lanceolata* Hochst. & Steud. In The Plant List 2016. http://www.theplantlist.org/tpl1.1/record/kew-2396402.

[44] Polhill P.M. (2005). Flora of Tropical East Africa: Santalaceae.

[45] Nickrent D. (2017). Status of the Genera Colpoon, Osyris and Rhoiacarpos in South Africa Molecular Phylogenetic Data. No. 1961. Bothalia Afr. Biodivers. Conserv..

[46] Fay M.F., Bennett J.R., Dixon K.W., Christenhusz M.J.M. (2010). Parasites, their relationships and the disintegration of Scrophulariaceae sensu lato. Curtis’s Bot. Mag..

[47] Mugula B.B. (2020). Field Surveys for Mapping Osyris Lanceolata in Karamoja, Sub-Region, Uganda. Field Work.

[48] Breitenbach F. (1963). The Indigenous Trees of Ethiopia.

[49] Bahadur K.K. (2018). Current status, distribution pattern and associations of Sandalwood (*Santalum album*) in Pyuthan District of Nepal. Clar. Int. Multidiscip. J..

[50] Harbaugh D.T., Baldwin B.G. (2007). Phylogeny and biogeography of the sandalwoods (Santalum, Santalaceae): Repeated dispersals throughout the Pacific. Am. J. Bot..

[51] Ndangalasi H.J., Mligo C., Mvungi E.F. (2014). Composition and Size Class Structure of Tree Species in Ihang’ana Forest Reserve, Mufindi District, Tanzania. Tanzan. J. Sci..

[52] Schueler L., Hemp A. (2016). Atlas of Pollen and Spores and Their Parent Taxa of Mt Kilimanjaro and Tropical East Africa. Quat. Int..

[53] (2019). Report on Africa Regional Meeting, CITES Tree Species Programme (CTSP) 10–15 March 2019, Dar Es Salaam, Tanzania. https://cites-tsp.org/wp-content/uploads/2020/02/CITES-Tree-Species-Programme-Regional-Meeting-for-Africa-11-to-15-March-2019-Dar-es-Salaam-Tanzania-%E2%80%93-Minutes..pdf.

[54] Ochanda K.V. (2009). Conservation and Management of Sandalwood Trees: (*Osyris lanceolata* Hochst & Steudel,) in Chyullu Hills Kibwezi District, Kenya. MSc. Thesis.

[55] Orwa C., Mutua A., Kindt R., Jamnadass R., Simons A. (2009). Agroforestree Database: A Tree Reference and Selection Guide. Version 4.

[56] Turini F.G., Steinert C., Heubl G., Bringmann G., Lombe B.K., Mudogo V., Meimberg H. (2014). Microsatellites facilitate species delimitation in Congolese *Ancistrocladus* (Ancistrocladaceae), a genus with pharmacologically potent naphthylisoquinoline alkaloids. Taxon.

[57] Meimberg H., Rischer H., Turini F.G., Chamchumroon V., Dreyer M., Sommaro M., Bringmann G., Heubl G. (2010). Evidence for species differentiation within the *Ancistrocladus tectorius* complex (Ancistrocladaceae) in Southeast Asia: A molecular approach. Plant Syst. Evol..

[58] Perrino E.V., Silletti G.N., Erben M., Wagensommer R.P. (2018). *Viola Cassinensis* Subsp. Lucana (Violaceae), a New Subspecies from the Lucanian Apennine, Southern Italy. Phyton Ann. Rei Bot..

[59] Kokwaro J.O. (2009). Osyris lanceolata. Medicinal Plants of East Africa.

[60] Tennakoon K.U., Cameron D.D. (2006). The anatomy of *Santalum album* (Sandalwood) haustoria. Can. J. Bot..

[61] Okubamichael D.Y., Griffiths M.E., Ward D. (2016). Host specificity in parasitic plants—Perspectives from mistletoes. AoB Plants.

[62] Bell T.L., Adams M. (2011). Attack on all fronts: Functional relationships between aerial and root parasitic plants and their woody hosts and consequences for ecosystems. Tree Physiol..

[63] Marvier M.A. (1996). Parasitic Plant-Host Interactions: Plant Performance and Indirect Effects on Parasite-Feeding Herbivores. Ecology.

[64] Nge F.J., Ranathunge K., Kotula L., Cawthray G., Lambers H. (2019). Strong host specificity of a root hemi-parasite (*Santalum acuminatum*) limits its local distribution: Beggars can be choosers. Plant Soil.

[65] Furuhashi T., Furuhashi K., Weckwerth W. (2011). The parasitic mechanism of the holostemparasitic plant *Cuscuta*. J. Plant Interact..

[66] Kuijt J. (1969). The Biology of Parasitic Flowering Plants.

[67] Watson D.M. (2009). Determinants of parasitic plant distribution: The role of host quality. Botany.

[68] Irving L.J., Cameron D.D. (2009). You are What You Eat: Interactions between Root Parasitic Plants and Their Hosts. Adv. Bot. Res..

[69] Yoder J.I. (1999). Parasitic plant responses to host plant signals: A model for subterranean plant–plant interactions. Curr. Opin. Plant Biol..

[70] Westwood J.H., Yoder J.I., Timko M.P., Depamphilis C.W. (2010). The evolution of parasitism in plants. Trends Plant Sci..

[71] Tomilov A., Tomilova N., Shin D.H., Jamison D., Torres M., Reagan R., Mcgray H., Horning T., Truong R., Nava A.J. (2006). Chemical Signalling between Plants. Chemical Ecology: From Gene to Ecosystem.

[72] Nilsson C.H., Svensson B.M. (1997). Host affiliation in two subarctic hemiparasitic plants: *Bartsia alpina* and *Pedicularis lapponica*. Écoscience.

[73] Matthies D., Egli P. (1999). Response of a root hemiparasite to elevated CO_2_ depends on host type and soil nutrients. Oecologia.

[74] Tennakoon K.U., Pate J.S. (1996). Heterotrophic gain of carbon from hosts by the xylem-tapping root hemiparasite *Olax phyllanthi* (Olacaceae). Oecologia.

[75] Vellend M. (2005). Species Diversity and Genetic Diversity: Parallel Processes and Correlated Patterns. Am. Nat..

[76] Miller D.L., Burt M.L., Rexstad E., Thomas L. (2013). Spatial models for distance sampling data: Recent developments and future directions. Methods Ecol. Evol..

[77] Gacheri N., Wanjala B.W., Jamnadass R., Muchugi A. (2016). Analysis of the Impact of Domestication of *Warburgia ugandensis* (Sprague) on Its Genetic Diversity Based on Amplified Fragment Length Polymorphism. Afr. J. Biotechnol..

[78] Ellegren H., Galtier N. (2016). Determinants of genetic diversity. Nat. Rev. Genet..

[79] Indrioko S., Ratnaningrum Y.W. (2015). Habitat Loss Caused Clonality, Genetic Diversity Reduction and Reproductive Failure in *Santalum album* (Santalaceae), an Endangered Endemic Species of Indonesia. Procedia Environ. Sci..

[80] Farwig N., Braun C., Böhning-Gaese K. (2007). Human disturbance reduces genetic diversity of an endangered tropical tree, *Prunus africana* (Rosaceae). Conserv. Genet..

[81] Zhang J., Wang X., Yao J., Li Q., Liu F., Yotsukura N., Krupnova T.N., Duan D. (2017). Effect of domestication on the genetic diversity and structure of *Saccharina japonica* populations in China. Sci. Rep..

[82] Graudal L., Aravanopoulos F., Bennadji Z., Changtragoon S., Fady B., Kjaer E., Loo J., Ramamonjisoa L., Vendramin G.G. (2014). Global to local genetic diversity indicators of evolutionary potential in tree species within and outside forests. For. Ecol. Manag..

[83] Frankham R., Ballou J.D., Briscoe D.A. (2002). Introduction to Conservation Genetics.

[84] Yang H., Zhang R., Jin G., Feng Z., Zhou Z. (2016). Assessing the Genetic Diversity and Genealogical Reconstruction of Cypress (*Cupressus funebris* Endl.) Breeding Parents Using SSR Markers. Forests.

[85] Vekemans X., Hardy O. (2004). New insights from fine-scale spatial genetic structure analyses in plant populations. Mol. Ecol..

[86] Petkova D., Novembre J., Stephens M. (2016). Visualizing spatial population structure with estimated effective migration surfaces. Nat. Genet..

[87] Volis S., Ormanbekova D., Shulgina I. (2016). Fine-scale spatial genetic structure in predominantly selfing plants with limited seed dispersal: A rule or exception?. Plant Divers..

[88] McQuillan R., Leutenegger A.-L., Abdel-Rahman R., Franklin C.S., Pericic M., Barac-Lauc L., Smolej-Narancic N., Janicijevic B., Polasek O., Tenesa A. (2008). Runs of Homozygosity in European Populations. Am. J. Hum. Genet..

[89] Szczecińska M., Sramko G., Wołosz K., Sawicki J. (2016). Genetic Diversity and Population Structure of the Rare and Endangered Plant Species *Pulsatilla patens* (L.) Mill in East Central Europe. PLoS ONE.

[90] Porth I., El-Kassaby Y.A. (2014). Assessment of the Genetic Diversity in Forest Tree Populations Using Molecular Markers. Diversity.

[91] Holliday J.A., Aitken S.N., Cooke J.E.K., Fady B., Gonzalez-Martinez S.C., Heuertz M., Jaramillo-Correa J.-P., Lexer C., Staton M., Whetten R.W. (2016). Advances in ecological genomics in forest trees and applications to genetic resources conservation and breeding. Mol. Ecol..

[92] Castoe T.A., Poole A.W., de Koning A.J., Jones K.L., Tomback D.F., Oyler-McCance S., Fike J.A., Lance S., Streicher J., Smith E.N. (2012). Rapid Microsatellite Identification from Illumina Paired-End Genomic Sequencing in Two Birds and a Snake. PLoS ONE.

[93] De Barba M., Miquel C., Lobréaux S., Quenette P.Y., Swenson J.E., Taberlet P. (2017). High-throughput microsatellite genotyping in ecology: Improved accuracy, efficiency, standardization and success with low-quantity and degraded DNA. Mol. Ecol. Resour..

[94] Neophytou C., Torutaeva E., Winter S., Meimberg H., Hasenauer H., Curto M. (2018). Analysis of microsatellite loci in tree of heaven (*Ailanthus altissima* (Mill.) Swingle) using SSR-GBS. Tree Genet. Genomes.

[95] Tibihika P.D., Curto M., Dornstauder-Schrammel E., Winter S., Alemayehu E., Waidbacher H., Meimberg H. (2019). Application of microsatellite genotyping by sequencing (SSR-GBS) to measure genetic diversity of the East African *Oreochromis niloticus*. Conserv. Genet..

[96] Vartia S., Villanueva-Cañas J.L., Finarelli J., Farrell E.D., Collins P.C., Hughes G., Carlsson J.E.L., Gauthier D.T., McGinnity P., Cross T.F. (2016). A novel method of microsatellite genotyping-by-sequencing using individual combinatorial barcoding. R. Soc. Open Sci..

[97] Šarhanová P., Pfanzelt S., Brandt R., Himmelbach A., Blattner F.R. (2018). SSR-seq: Genotyping of microsatellites using next-generation sequencing reveals higher level of polymorphism as compared to traditional fragment size scoring. Ecol. Evol..

[98] Parchman T.L., Jahner J.P., Uckele K.A., Galland L.M., Eckert A.J. (2018). RADseq approaches and applications for forest tree genetics. Tree Genet. Genomes.

[99] Campbell N.R., Harmon S.A., Narum S.R. (2015). Genotyping-in-Thousands by sequencing (GT-seq): A cost effective SNP genotyping method based on custom amplicon sequencing. Mol. Ecol. Resour..

[100] Dennenmoser S., Vamosi S.M., Nolte A.W., Rogers S.M. (2017). Adaptive genomic divergence under high gene flow between freshwater and brackish-water ecotypes of prickly sculpin (*Cottus asper*) revealed by Pool-Seq. Mol. Ecol..

[101] Fox J.E.D. (1997). Why is Santalum Spicatum Common Near Granite Rocks?. J. R. Soc. West. Aust..

[102] Pfenninger M., Salinger M., Haun T., Feldmeyer B. (2011). Factors and processes shaping the population structure and distribution of genetic variation across the species range of the freshwater snail *radix balthica* (Pulmonata, Basommatophora). BMC Evol. Biol..

[103] Amundrud S.L. (2020). Abiotic and Biotic Processes Shape Species Distributions and Ecological Communities across Spatial Scales. Ph.D. Thesis.

[104] Kamondo B., Giathi G., Osore C., Machua J., Kagunyu L., Wafula A., Bala P., Njuguna J., Wakori S., Maingi F. (2014). Growing of East African Sandalwood: Guidelines for Tree Growers.

[105] Mortelliti A., Amori G., Boitani L. (2010). The role of habitat quality in fragmented landscapes: A conceptual overview and prospectus for future research. Oecologia.

[106] Laurance W.F. (2010). Habitat destruction: Death by a thousand cuts. Conserv. Biol. All.

[107] Chase J.M., Blowes S.A., Knight T.M., Gerstner K., May F. (2020). Ecosystem decay exacerbates biodiversity loss with habitat loss. Nature.

[108] McKinnell F.H., Levinson J. (2008). WA Sandalwood Industry Development Plan 2008–2020.

[109] Okiror P., Chono J., Nyamukuru A., Lwanga J.S., Sasira P., Diogo P. (2012). Variation in Woody Species Abundance and Distribution in and around Kibale National Park, Uganda. Int. Sch. Res. Not. For..

[110] Wiegand K., Jeltsch F., Ward D. (1999). Analysis of the population dynamics of Acacia trees in the Negev desert, Israel with a spatially-explicit computer simulation model. Ecol. Model..

[111] Lindenmayer D.B., Laurance W.F. (2016). The ecology, distribution, conservation and management of large old trees. Biol. Rev..

[112] Shyaula S. (2012). A review on genus Osyris: Phytochemical constituents and traditional uses. J. Nat. Pharm..

[113] Njoroge G.N., Bussmann R.W. (2006). Diversity and utilization of antimalarial ethnophytotherapeutic remedies among the Kikuyus (Central Kenya). J. Ethnobiol. Ethnomed..

[114] Moy R.L., Levenson C. (2017). Sandalwood Album Oil as a Botanical Therapeutic in Dermatology. J. Clin. Aesthet. Dermatol..

[115] Dwivedi C., Abu-Ghazaleh A. (1997). Chemopreventive effects of sandalwood oil on skin papillomas in mice. Eur. J. Cancer Prev..

[116] Liu X., Gao Y., Khan S., Duan G., Chen A., Ling L., Zhao L., Liu Z., Wu X. (2008). Accumulation of Pb, Cu, and Zn in native plants growing on contaminated sites and their potential accumulation capacity in Heqing, Yunnan. J. Environ. Sci..

[117] Jain R., Nair S. (2019). Sandalwood Oil for the Chemoprevention of Skin Cancer: Mechanistic Insights, Anti-inflammatory, and In Vivo Anticancer Potential. Curr. Pharmacol. Rep..

[118] Coppen J. (1995). Flavours and Fragrances of Plant Origin.

[119] Hemp A., Hemp C., Winter J.C. (2009). Environment and Worldview: The Chagga Homegardens.

[120] Bhowmik D., Biswas D., Kumar K.P.S. (2011). Recent Aspect of Ethnobotanical Application and Medicinal Properties of Traditional Indian Herbs *Santalum album*. Int. J. Chem. Res..

[121] Akbar S. (2020). Santalum album L. (Santalaceae). Handbook of 200 Medicinal Plants.

[122] Wang C., Kim S.-W. (2015). Shaking up ancient scents: Insights into santalol synthesis in engineered *Escherichia coli*. Process. Biochem..

[123] Cogoni D., Fenu G., Dessì C., Deidda A., Giotta C., Piccitto M., Bacchetta G. (2021). Importance of Plants with Extremely Small Populations (PSESPs) in Endemic-Rich Areas, Elements Often Forgotten in Conservation Strategies. Plants.

[124] Sulis E., Bacchetta G., Cogoni D., Fenu G. (2021). From Global to Local Scale: Where is the Best for Conservation Purpose?. Biodivers. Conserv..

[125] Fenu G., Bacchetta G., Christodoulou C.S., Cogoni D., Fournaraki C., Gian Pietro G.D.G., Gotsiou P., Kyratzis A., Piazza C., Vicens M. (2020). A Common Approach to the Conservation of Threatened Island Vascular Plants: First Results in the Mediterranean Basin. Diversity.

[126] WFO (2021). Osyris lanceolata Hochst. & Steud. http://www.worldfloraonline.org/taxon/wfo-0000388245.

[127] Missouri Botanical Garden. https://tropicos.org.

